# Effects of Continuous Positive Airway Pressure on Surrogate, Intermediate, and Functional Cardiovascular Outcomes in Obstructive Sleep Apnea: An Umbrella Review of Systematic Reviews and Meta-Analyses

**DOI:** 10.3390/medicina62081456

**Published:** 2026-07-27

**Authors:** Jeta Bunjaku, Premtim Rashiti, Arber Lama, Genta Bunjaku, Rozafa Koliqi

**Affiliations:** 1 Department of Pulmonology, University Clinical Center of Kosovo, 10 000 Prishtina, Kosovo; jeta.bunjaku@uni-pr.edu; 2 Faculty of Medicine, University of Prishtina ‘Hasan Prishtina’, 10 000 Prishtina, Kosovo; premtim.rashiti@uni-pr.edu; 3 Evidence Synthesis Group, 10 000 Prishtina, Kosovo; lamaarber@gmail.com (A.L.); gentabunjaku123@gmail.com (G.B.); 4 Emergency Clinic, University Clinical Center of Kosovo, 10 000 Prishtina, Kosovo; 5 Institute of Forensic Medicine, University Clinical Center of Kosovo, 10 000 Prishtina, Kosovo; 6 Department of Clinical Pharmacy and Biopharmacy, Pharmacy Division, Faculty of Medicine, University of Prishtina ‘Hasan Prishtina’, 10 000 Prishtina, Kosovo

**Keywords:** obstructive sleep apnea, CPAP, cardiovascular outcomes, umbrella review, meta-analysis

## Abstract

*Background and Objectives*: Obstructive sleep apnea (OSA) is a prevalent sleep-related breathing disorder associated with increased cardiovascular morbidity and mortality. Continuous positive airway pressure (CPAP) is the standard treatment for OSA; however, its effects on cardiovascular outcomes remain incompletely established. This umbrella review synthesized and critically appraised the evidence regarding the effects of CPAP on surrogate, intermediate, and functional cardiovascular outcomes in adults with OSA. *Materials and Methods*: An umbrella review of systematic reviews and meta-analyses evaluating CPAP therapy in adults with OSA was conducted. A comprehensive literature search was performed in PubMed, Embase, and the Cochrane Library from database inception to 28 February 2026. Eligible reviews assessed cardiovascular outcomes, including blood pressure, cardiac function, autonomic function, cardiovascular biomarkers, and functional cardiovascular measures. Methodological quality was evaluated using AMSTAR 2, and the certainty of evidence for the main outcomes was assessed using the GRADE framework. *Results*: Thirty-eight systematic reviews and meta-analyses were included. CPAP therapy was consistently associated with reductions in systolic blood pressure (mean difference [MD] −4.8 mmHg) and diastolic blood pressure (MD −3.0 mmHg), with additional improvements in 24 h ambulatory blood pressure. Favorable effects were also observed for left ventricular ejection fraction (MD 3.27), left ventricular diastolic function (weighted mean difference [WMD] 0.22), and sympathetic nervous system activity, reflected by lower circulating noradrenaline levels (standardized mean difference [SMD] −1.10) in randomized controlled trials. Improvements in BNP/NT-proBNP concentrations and New York Heart Association functional class were reported among patients with heart failure, whereas no significant effect was observed on body mass index. The certainty of evidence ranged from low to moderate, and methodological confidence of the included reviews was predominantly moderate, low, or critically low. *Conclusions*: CPAP therapy was associated with improvements in several surrogate, intermediate, and functional cardiovascular outcomes in adults with OSA, particularly blood pressure, left ventricular function, and sympathetic activity. However, the certainty of evidence ranged from low to moderate, and substantial heterogeneity and methodological limitations warrant cautious interpretation. Current evidence remains insufficient to demonstrate a consistent reduction in major adverse cardiovascular events or cardiovascular mortality. Further high-quality randomized controlled trials with longer follow-up are needed to clarify the long-term cardiovascular impact of CPAP therapy.

## 1. Introduction

Obstructive sleep apnea (OSA) is a common sleep-related breathing disorder characterized by recurrent upper airway obstruction during sleep, intermittent hypoxia, and sleep fragmentation [1,2,3]. High body mass index (BMI), increasing age, and male sex are recognized as the main risk factors for OSA [4]. OSA affects a substantial proportion of the adult population worldwide, with an estimated prevalence ranging from 9% to 38%, depending on population characteristics and criteria [5,6,7].

A large and growing body of evidence has demonstrated a strong association between OSA and adverse cardiovascular outcomes, including hypertension, coronary artery disease, heart failure, and cardiac arrhythmias [8,9,10,11,12,13,14,15,16,17,18,19,20]. Cardiac structure and electrophysiology may be affected in patients with OSA through sympathetic activation, left ventricular remodeling, vagal stimulation, and systemic inflammation, contributing to the development of atrial fibrillation, ventricular arrhythmias, and bradyarrhythmias [14,17,18,19,20]. The underlying pathophysiological mechanisms linking OSA to cardiovascular disease are complex and multifactorial, involving intermittent hypoxia, oxidative stress, systemic inflammation, endothelial dysfunction, and increased sympathetic nervous system activity [21,22,23].

Continuous positive airway pressure (CPAP) remains the gold-standard treatment for OSA. CPAP maintains upper airway patency during sleep, normalizes the apnea–hypopnea index (AHI), improves oxygen saturation, and reduces excessive daytime sleepiness [24,25]. Although the benefits of CPAP on sleep-related symptoms are well established, its effects on cardiovascular outcomes remain less certain [26,27,28,29]. Randomized controlled trials and observational studies have reported improvements in several surrogate and intermediate cardiovascular outcomes, such as blood pressure and cardiac function, whereas evidence for hard cardiovascular endpoints, including major adverse cardiovascular events and mortality, remains limited and inconsistent [30,31].

Several systematic reviews and meta-analyses have evaluated the cardiovascular effects of CPAP therapy in patients with OSA. However, these reviews have primarily focused on individual cardiovascular outcomes or specific patient populations and differ considerably in their eligibility criteria, study designs, cardiovascular outcome domains, and methodological quality. Consequently, the available evidence remains fragmented and, in some cases, inconsistent, limiting a comprehensive understanding of the overall cardiovascular effects of CPAP therapy.

To address this knowledge gap, the present umbrella review systematically synthesizes evidence across multiple predefined cardiovascular outcome domains, including surrogate, intermediate, and functional outcomes. In addition, it critically appraises the methodological quality of the included reviews using the AMSTAR 2 tool and evaluates the certainty of evidence for the main outcomes using the GRADE approach. By integrating findings from published systematic reviews and meta-analyses into a single comprehensive evidence synthesis, this umbrella review provides an overarching assessment of the available evidence, identifies areas of consistent findings, highlights uncertainties, and outlines priorities for future research.

Therefore, the aim of this umbrella review was to comprehensively synthesize and critically appraise the available evidence regarding the effects of CPAP therapy on surrogate, intermediate, and functional cardiovascular outcomes in adults with obstructive sleep apnea.

## 2. Materials and Methods

### 2.1. Protocol and Reporting Guidelines

A predefined methodological protocol was developed before study selection and data extraction, specifying the review objectives, eligibility criteria, cardiovascular outcome domains, the literature search strategy, quality assessment methods, and evidence synthesis approach. This umbrella review was conducted and reported in accordance with the PRISMA 2020 Statement [32] and established methodological recommendations for umbrella reviews [33]. Although the review protocol was not prospectively registered in PROSPERO, all methodological decisions were defined a priori and remained unchanged throughout the review process.

### 2.2. Literature Search

A comprehensive systematic literature search was performed in PubMed, Embase, and the Cochrane Library from database inception until 28 February 2026. Search terms combined concepts related to obstructive sleep apnea, continuous positive airway pressure, and cardiovascular outcomes, including blood pressure, cardiac function, heart rate variability, endothelial function, biomarkers, and cardiovascular events. The search was limited to studies involving humans and published in English from 2010 onward. Reviews published in 2025 or early 2026 were eligible if they had been electronically published ahead of print before the predefined search end date. In addition, the reference lists of all eligible reviews were manually screened to identify additional relevant studies. The complete search strategies for each database are provided in Supplementary Table S1.

### 2.3. Eligibility Criteria

Studies were eligible if they were systematic reviews, with or without meta-analysis, evaluating the effects of continuous positive airway pressure (CPAP) therapy on predefined surrogate, intermediate and functional cardiovascular outcomes in adults with obstructive sleep apnea (OSA). Reviews including randomized controlled trials, observational studies, or mixed study designs were considered eligible. Exclusion criteria comprised narrative reviews, scoping reviews without systematic methodology, primary studies, case reports, case series, editorials, letters, commentaries, study protocols, conference abstracts without full text, duplicate publications, animal studies, and reviews that did not evaluate the effects of CPAP on the predefined surrogate, intermediate, or functional cardiovascular outcome domains.

### 2.4. Study Selection and Data Extraction

Two reviewers independently screened titles, abstracts, and full texts for eligibility. Disagreements were resolved through discussion or adjudication by a third reviewer. Data extraction was performed independently using a standardized spreadsheet, and discrepancies were resolved by consensus. Extracted data included first author, year of publication, study design, number of included studies, sample size, population characteristics, CPAP exposure, reported cardiovascular outcomes, effect estimates, confidence intervals, *p*-values, heterogeneity statistics, and information relevant to publication bias.

#### 2.4.1. Classification of Cardiovascular Outcome Domains

To improve the consistency and clinical interpretability of the evidence synthesis, cardiovascular outcomes were classified a priori into predefined outcome domains according to their clinical relevance and underlying pathophysiological characteristics.

Surrogate cardiovascular outcomes comprised physiological, hemodynamic, endothelial, vascular, and biomarker-based measures reflecting early cardiovascular alterations, including systolic and diastolic blood pressure, endothelial function, circulating catecholamine levels, cardiovascular biomarkers, arterial stiffness indices, and related surrogate measures.

Intermediate cardiovascular outcomes included structural, functional, and electrophysiological cardiac changes assessed by imaging or physiological investigations, such as left ventricular ejection fraction (LVEF), left ventricular diastolic function, myocardial strain, pulmonary artery pressure, pulmonary vascular resistance, atrial remodeling, heart rate variability (HRV), atrial fibrillation recurrence, and bradyarrhythmia-related outcomes.

Functional cardiovascular outcomes referred to measures of patients’ functional status and cardiovascular performance, including New York Heart Association (NYHA) functional class and other exercise-related functional measures, where reported.

#### 2.4.2. Outcome-Level Evidence Synthesis

When multiple systematic reviews reported meta-analytic estimates for the same cardiovascular outcome, representative effect estimates were selected using a predefined hierarchical approach. Priority was given to reviews with the highest methodological quality according to AMSTAR 2, followed by the largest cumulative sample size and the most comprehensive quantitative synthesis. Where conflicting findings were identified among high-quality reviews, results were interpreted according to CPAP adherence (e.g., ≥4 h/night vs. <4 h/night), duration of therapy, follow-up period, and characteristics of the included populations. This approach minimized potential bias arising from overlapping evidence and ensured that the final outcome synthesis reflected the most methodologically robust and clinically representative evidence available.

Outcome domains that were reported infrequently, lacked pooled quantitative estimates, or could not be synthesized consistently across multiple systematic reviews because of substantial methodological or clinical heterogeneity were summarized narratively rather than prioritized in the main quantitative outcome synthesis. This approach ensured that the summary tables reflected the most robust and consistently reported cardiovascular outcomes while preserving all relevant evidence within the narrative synthesis.

This predefined hierarchical strategy also served to minimize potential double-counting of evidence arising from overlapping systematic reviews.

### 2.5. Methodological Quality and Certainty of Evidence

The methodological quality of the included systematic reviews and meta-analyses was independently assessed by two reviewers using AMSTAR 2 (A Measurement Tool to Assess Systematic Reviews 2), which evaluates 16 methodological domains and classifies overall confidence as high, moderate, low, or critically low [33,34]. Although all 16 AMSTAR 2 domains were assessed, particular emphasis was placed on the critical domains, including protocol registration, comprehensiveness of the literature search, risk-of-bias assessment, and justification of excluded studies.

The certainty of evidence for the main cardiovascular outcome domains was assessed using the GRADE (Grading of Recommendations Assessment, Development and Evaluation) framework [35]. Formal GRADE assessment was restricted to blood pressure, heart rate variability, and cardiovascular biomarkers because these outcome domains were supported by multiple quantitative meta-analyses and provided sufficient and comparable evidence for a structured assessment of certainty. Other cardiovascular outcomes were not formally graded because they were reported by only one or a limited number of systematic reviews, lacked sufficiently comparable pooled estimates, or exhibited substantial clinical and methodological heterogeneity that precluded a reliable domain-level certainty assessment. These outcomes were therefore synthesized narratively. Certainty ratings were based on the five GRADE domains: risk of bias, inconsistency, indirectness, imprecision, and publication bias. Downgrading decisions were applied whenever serious or very serious concerns were identified within these domains, in accordance with GRADE guidance.

### 2.6. Overlap of Primary Studies

Because umbrella reviews synthesize evidence from previously published systematic reviews and meta-analyses, overlap of primary studies is an inherent methodological consideration that may influence the interpretation of findings.

We evaluated the feasibility of performing a formal Corrected Covered Area (CCA) analysis. However, this umbrella review synthesized evidence across multiple predefined cardiovascular outcome domains (surrogate, intermediate, and functional outcomes) rather than a single clinical endpoint. Consequently, the included systematic reviews frequently evaluated different outcomes and reported outcome-specific meta-analyses. In addition, several reviews did not provide complete lists of primary studies, preventing the construction of reliable citation matrices across all outcome domains. Therefore, a formal CCA analysis was not considered appropriate for the overall evidence base, as a single overall CCA would have provided a potentially misleading estimate of overlap.

Instead, overlap was assessed descriptively by comparing publication years, search periods, eligibility criteria, intervention characteristics, cardiovascular outcomes, and reported primary studies whenever available. To minimize the risk of double-counting evidence, representative effect estimates were selected using the predefined hierarchical approach described above, prioritizing reviews with the highest AMSTAR 2 rating, largest cumulative sample size, and most comprehensive quantitative synthesis. Pooled estimates from overlapping systematic reviews were not combined, and only one representative meta-analysis was included for each cardiovascular outcome. Although some overlap among primary studies cannot be excluded, its potential influence was considered during evidence synthesis and interpretation and is acknowledged as an inherent limitation of this umbrella review.

### 2.7. Data Synthesis

A narrative synthesis supplemented by outcome-level quantitative evidence synthesis was conducted according to the predefined surrogate, intermediate, and functional cardiovascular outcome domains described above. Because of variability in study populations, cardiovascular outcome definitions, follow-up duration, CPAP adherence, and review methodology, no additional meta-analysis was performed. Findings were synthesized by considering the direction and magnitude of effect estimates, statistical significance, heterogeneity, methodological quality (AMSTAR 2), and certainty of evidence (GRADE). Where multiple systematic reviews reported the same cardiovascular outcome, the predefined hierarchical selection strategy described in Section 2.4.2 was applied to minimize overlap and ensure that the most methodologically robust evidence contributed to the final synthesis.

## 3. Results

### 3.1. Study Selection

**Figure 1:**
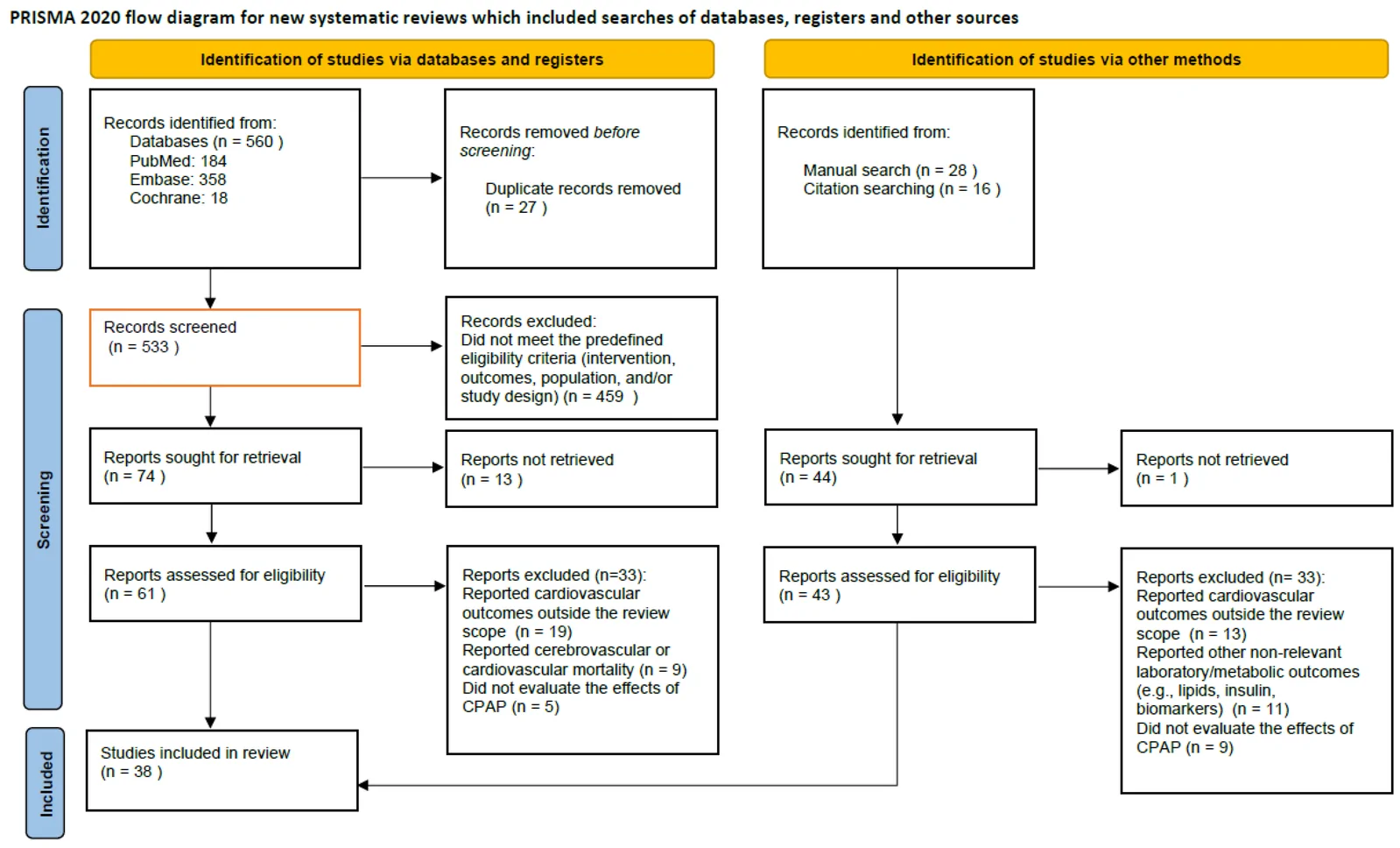
PRISMA 2020 flow diagram of the study selection process.

A total of 560 records were identified through database searching, and 44 additional records were identified through other methods (28 through manual searching and 16 through citation searching). After removal of 27 duplicate records, 533 records were screened by title and abstract, of which 459 were excluded. Seventy-four reports identified through database searching were sought for retrieval, and 13 could not be retrieved, leaving 61 reports for full-text eligibility assessment. In addition, 44 reports identified through other methods were sought for retrieval, of which one could not be retrieved, leaving 43 reports for full-text eligibility assessment. Following full-text review, 66 reports were excluded for prespecified reasons, and 38 systematic reviews and meta-analyses met the eligibility criteria and were included in this umbrella review [27,36,37,38,39,40,41,42,43,44,45,46,47,48,49,50,51,52,53,54,55,56,57,58,59,60,61,62,63,64,65,66,67,68,69,70,71,72]. The study selection process is illustrated in Figure 1 using the PRISMA 2020 flow diagram.

### 3.2. Characteristics of Included Reviews

The 38 included systematic reviews and meta-analyses were published between 2012 and 2026. The number of primary studies included in each review ranged from 4 to 52, while reported sample sizes ranged from 167 to 10,104 participants. Most reviews included adults with obstructive sleep apnea (OSA), including both general OSA populations and patients with cardiovascular comorbidities. The cardiovascular outcomes evaluated encompassed blood pressure, heart rate variability, cardiac function, vascular outcomes, cardiovascular biomarkers, cardiovascular risk, and broader cardiovascular outcomes. Detailed characteristics of the included reviews are presented in Table 1.

### 3.3. Methodological Quality

**Figure 2:**
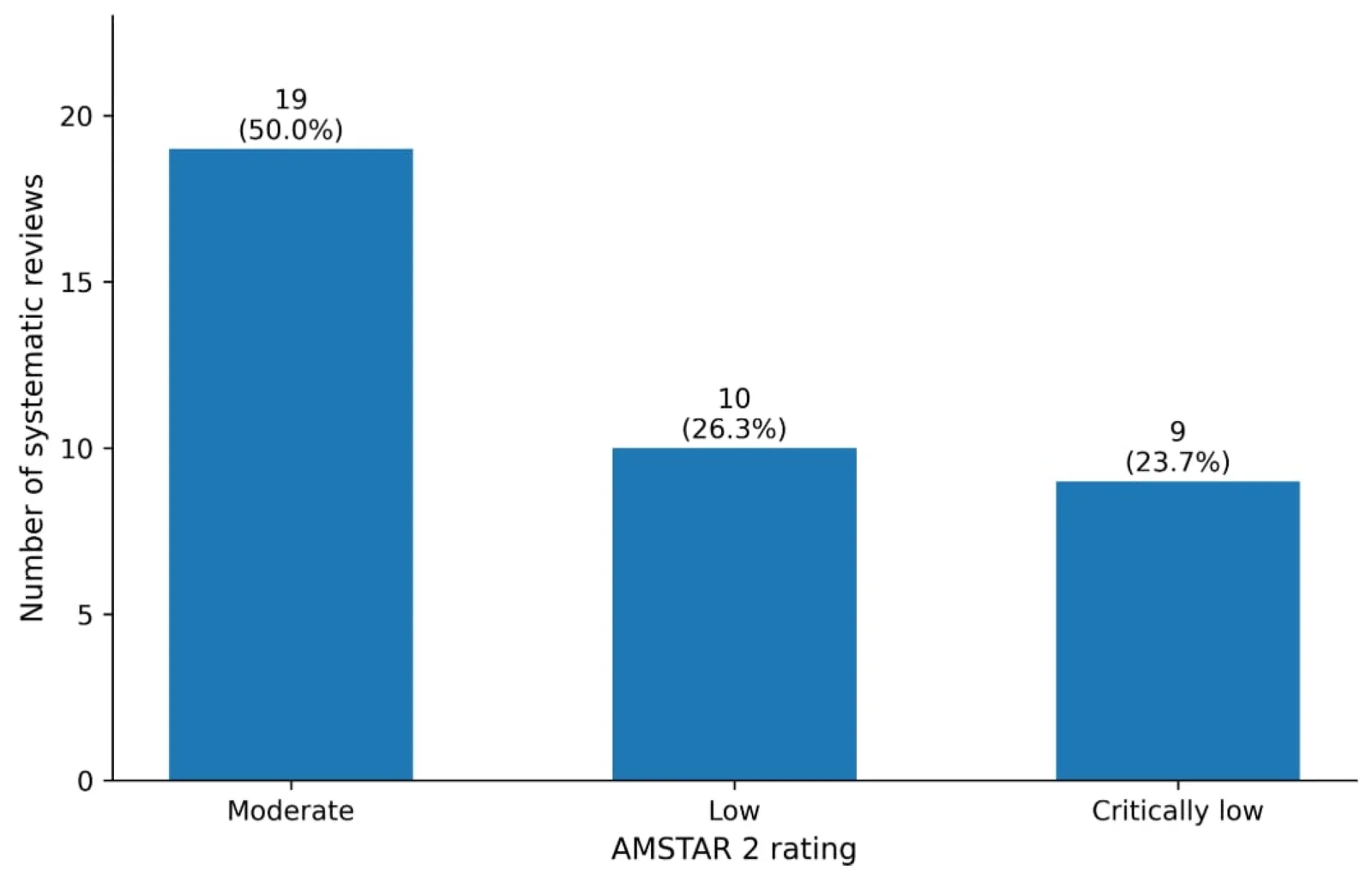
Distribution of the overall methodological confidence of the included systematic reviews according to the AMSTAR 2 overall confidence rating.

According to the AMSTAR 2 overall confidence rating, 19 reviews (50.0%) were classified as moderate, 10 (26.3%) as low, and 9 (23.7%) as critically low methodological confidence (Table 2 and Figure 2). No review was rated as high confidence. Common methodological limitations included lack of protocol registration, incomplete assessment of risk of bias, inadequate reporting of excluded studies, and limited evaluation of publication bias. These limitations were taken into account when interpreting the pooled cardiovascular evidence.

### 3.4. Effects of CPAP on Cardiovascular Outcomes

The evidence synthesis demonstrated that CPAP therapy was associated with beneficial effects on several cardiovascular outcomes. The most consistent benefits were observed for blood pressure reduction, with significant decreases in systolic and diastolic blood pressure, including 24 h and nocturnal measurements. CPAP was also associated with improvements in left ventricular ejection fraction and left ventricular diastolic function. In addition, treatment reduced circulating noradrenaline levels and was associated with improvements in selected markers of autonomic function, although findings for heart rate variability were not consistent across all parameters. Among patients with heart failure, CPAP was associated with lower BNP/NT-proBNP levels and improved NYHA functional class. No significant effect was observed on body mass index. Overall, these findings suggest that CPAP primarily improves surrogate cardiovascular outcomes, whereas the magnitude and consistency of benefit vary across different outcome domains because of heterogeneity across studies, small sample sizes, and limited reporting for some outcomes. The quantitative cardiovascular outcomes are summarized in Table 3.

### 3.5. Certainty of Evidence

**Figure 3:**
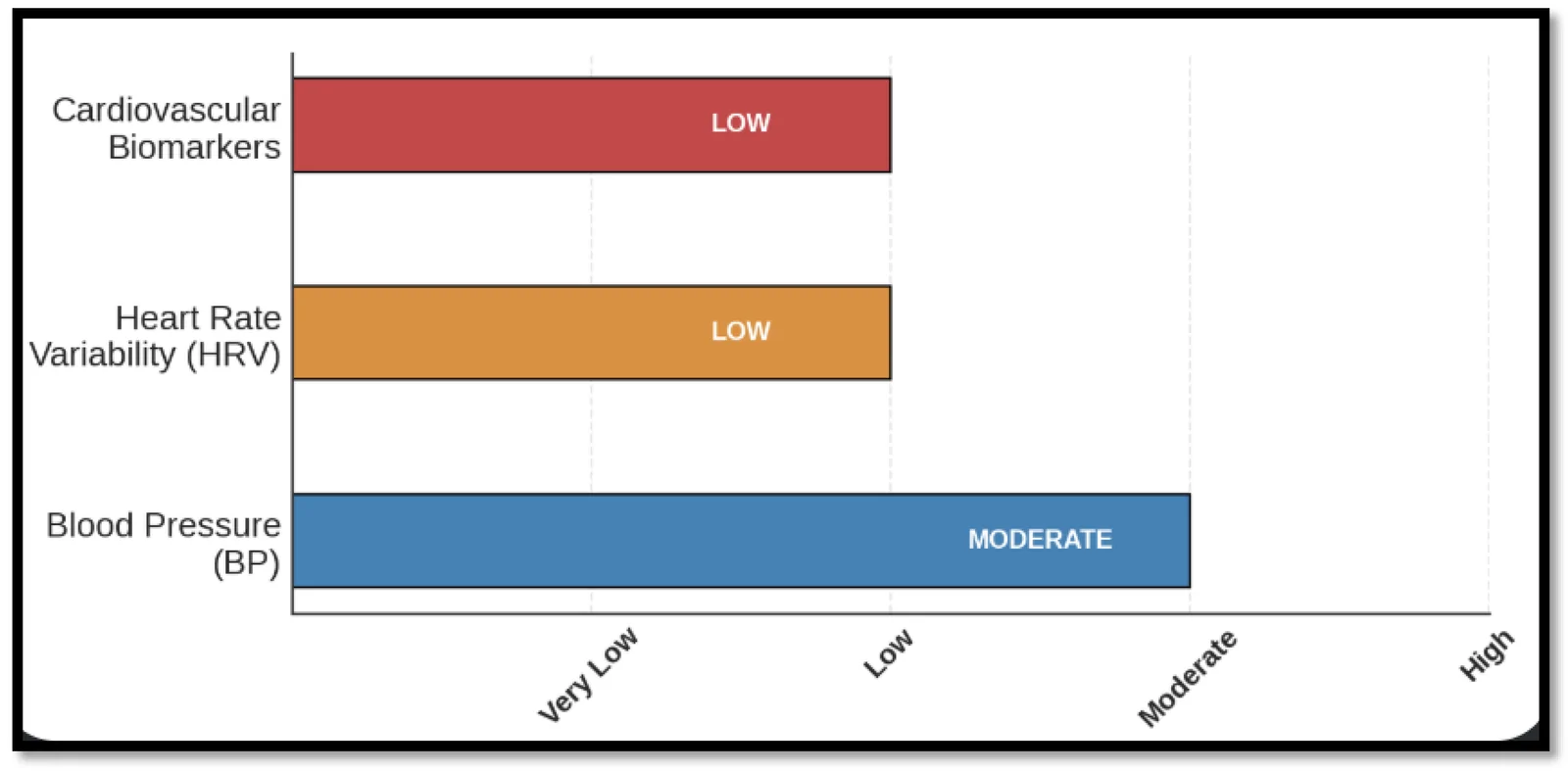
Consolidated GRADE evidence profile for the main cardiovascular outcomes.

The certainty of evidence ranged from low to moderate across the evaluated cardiovascular outcomes. Blood pressure outcomes were rated as moderate-certainty evidence, reflecting consistent reductions across multiple meta-analyses despite substantial statistical heterogeneity. In contrast, evidence for heart rate variability and cardiovascular biomarkers was rated as low certainty because of inconsistency between studies, limited sample sizes, and incomplete outcome reporting. The certainty-of-evidence assessments for the main cardiovascular outcomes are summarized in Table 4, and the consolidated GRADE evidence profile is presented in Figure 3.

## 4. Discussion

This umbrella review synthesized evidence from 38 systematic reviews and meta-analyses evaluating the effects of continuous positive airway pressure (CPAP) therapy on surrogate, intermediate, and functional cardiovascular outcomes in adults with obstructive sleep apnea (OSA). Overall, the findings indicate that CPAP provides the most consistent cardiovascular benefits for blood pressure reduction, improvement of left ventricular function, and attenuation of sympathetic nervous system activity. In contrast, evidence regarding heart rate variability, cardiovascular biomarkers, and long-term major adverse cardiovascular events remains less consistent. These findings suggest that CPAP exerts its greatest cardiovascular benefits on surrogate and functional outcomes, whereas evidence regarding hard cardiovascular endpoints remains inconclusive despite recent large randomized trials and meta-analyses [26,27,28,29,30,31].

The most robust finding across the included evidence was the reduction in blood pressure. Moderate-certainty evidence demonstrated consistent reductions in both systolic and diastolic blood pressure, including 24 h ambulatory and nocturnal blood pressure measurements. Although the magnitude of these reductions was generally modest, even small decreases in blood pressure may translate into clinically meaningful reductions in cardiovascular risk at the population level. These findings are consistent with previous systematic reviews and meta-analyses reporting beneficial effects of CPAP on blood pressure among patients with OSA [37,42,43,44,46,47,48,49,51,52,54,55,58,65,68,70]. Nevertheless, considerable statistical heterogeneity observed across several analyses suggests that treatment effects are likely influenced by differences in baseline blood pressure, OSA severity, cardiovascular comorbidities, CPAP adherence, and duration of follow-up.

Beyond blood pressure, CPAP demonstrated favorable effects on cardiac structure and function. Improvements in left ventricular ejection fraction, left ventricular diastolic function, BNP/NT-proBNP concentrations, and NYHA functional class were particularly evident among patients with heart failure. These findings are biologically plausible because intermittent hypoxia, oxidative stress, endothelial dysfunction, and chronic sympathetic activation contribute to adverse cardiac remodeling and impaired myocardial performance in OSA [8,21,22,23,31]. By eliminating recurrent upper airway obstruction and reducing nocturnal hypoxemia, CPAP may decrease cardiac afterload, improve myocardial oxygen supply-demand balance, and attenuate neurohumoral activation, thereby contributing to improved cardiac performance. The observed improvements in functional cardiac measures support the hypothesis that patients with established cardiovascular disease may derive greater benefit from CPAP than unselected OSA populations [12,13,36,39,53,56,63,66,72].

Another important finding was the reduction in circulating noradrenaline levels following CPAP therapy, supporting previous evidence that treatment attenuates chronic sympathetic nervous system overactivity, one of the principal mechanisms linking OSA with hypertension, arrhythmias, and cardiovascular disease [8,14,17,18,19,20,37]. In contrast, heart rate variability findings were less consistent. Although improvements were observed for selected HRV indices, other parameters showed no statistically significant changes, resulting in low-certainty evidence. This inconsistency likely reflects methodological differences in HRV assessment, relatively small sample sizes, variation in CPAP adherence, and heterogeneity in disease severity across the included studies.

Interestingly, CPAP showed no significant effect on body mass index. This finding is expected because CPAP primarily addresses upper airway obstruction and sleep-disordered breathing rather than the underlying metabolic determinants of obesity. Consequently, cardiovascular risk reduction associated with CPAP is unlikely to be mediated through weight loss alone, emphasizing the importance of combining CPAP therapy with lifestyle modification, weight management, and optimal treatment of cardiovascular risk factors whenever appropriate [4,10,11,24,25].

The clinical interpretation of these findings should remain cautious. Although CPAP consistently improves several surrogate and functional cardiovascular outcomes, large randomized controlled trials have reported inconsistent effects on major adverse cardiovascular events and cardiovascular mortality [26,27,28,29,30,31,41,61,69,73]. Several explanations have been proposed, including suboptimal CPAP adherence, inclusion of minimally symptomatic patients, relatively short follow-up periods, and the multifactorial nature of cardiovascular disease in patients with OSA [26,31]. Therefore, while CPAP clearly improves important physiological and functional cardiovascular parameters, current evidence remains insufficient to conclude that these improvements consistently translate into reductions in long-term cardiovascular events.

### Strengths and Limitations

This umbrella review has several limitations that should be acknowledged. First, although a predefined methodological protocol was established before study selection and data extraction, the review was not prospectively registered in PROSPERO, which may have reduced methodological transparency. Second, despite applying a predefined hierarchical approach to minimize duplication of evidence, some overlap of primary studies across the included systematic reviews cannot be excluded because a formal Corrected Covered Area (CCA) analysis was not feasible. Third, considerable clinical and methodological heterogeneity across the included reviews—including differences in patient populations, cardiovascular outcomes, CPAP adherence, follow-up duration, and study design—limited direct comparisons between studies and contributed to variability in the reported effect estimates. Finally, the conclusions of this umbrella review remain dependent on the methodological quality and reporting of the included systematic reviews, many of which were rated as moderate, low, or critically low confidence according to AMSTAR 2.

Despite these limitations, this review has several important strengths. It provides a comprehensive synthesis of evidence across multiple predefined cardiovascular outcome domains using a transparent and predefined methodology. The review was conducted in accordance with the PRISMA 2020 Statement, incorporated a comprehensive literature search, and systematically evaluated methodological quality using AMSTAR 2 together with certainty of evidence using the GRADE framework. These methodological features strengthen the transparency, reproducibility, and overall robustness of the evidence synthesis.

## 5. Conclusions

CPAP therapy is associated with clinically meaningful improvements in several surrogate, intermediate, and functional cardiovascular outcomes in adults with OSA, particularly blood pressure, left ventricular function, and sympathetic activity. However, the certainty of evidence ranged from low to moderate, and substantial methodological heterogeneity remains across the available literature. Although these findings support the role of CPAP as the cornerstone treatment for OSA with potential cardiovascular benefits, current evidence remains insufficient to establish a consistent reduction in major adverse cardiovascular events or cardiovascular mortality. Future well-designed randomized controlled trials with longer follow-up and standardized cardiovascular outcome reporting are needed to clarify the long-term cardiovascular impact of CPAP therapy and to identify the patient populations most likely to derive clinically meaningful benefit.

## Figures and Tables

**Table 1:** Characteristics of included systematic reviews and meta-analyses.

Author	Year	Design	Studies	Sample	Main Outcomes
Feng et al. [36]	2023	Systematic review and meta-analysis	n = 7 (RCT = 2; prospective = 5)	473	Left ventricular diastolic function
Green et al. [37]	2021	Meta-analysis	n = 38 (RCT = 14; cohort = 24)	1123	24 h urinary Noradrenaline
Guo et al. [27]	2016	Meta-analysis	n = 18 (RCTs)	4146	24 h Systolic BP
Guo et al. [38]	2018	Meta-analysis	n = 11 (cohort)	260	Apnea–Hypopnea Index (AHI)
Han et al. [39]	2021	Systematic review and meta-analysis	n = 11	392	Left Ventricular Ejection Fraction (LVEF)
Itaya et al. [40]	2025	Systematic review and meta-analysis	n = 11	1536	Atrial fibrillation recurrence (overall)
Khan et al. [41]	2017	Meta-analysis	n = 7	4268	SBP
Kou et al. [42]	2022	Meta-analysis	n = 10	606	Office SBP
Labarca et al. [43]	2021	Systematic review	n = 6	479	24 h SBP
Lei et al. [44]	2017	Meta-analysis	n = 8	1231	Mean 24 h SBP (ABPM)
Li et al. [45]	2023	Meta-analysis	n = 28	1948	Atrial fibrillation (AF)
Montesi et al. [46]	2012	Systematic review	n = 10	1148	Diurnal SBP
Pengo et al. [47]	2020	Meta-analysis	n = 7	794	SBP
Hu et al. [49]	2015	Meta-analysis	n = 16	1166	24 h SBP change
Schein et al. [51]	2014	Meta-analysis	n = 19	1904	Office SBP
Sun et al. [72]	2013	Meta-analysis	n = 6	181	LVEF (overall)
Sun et al. [53]	2014	Meta-analysis	n = 12	1720	Pulmonary arterial pressure (PAP: mPAP/PASP)
Tadic et al. [56]	2022	Meta-analysis	n = 12	718	Left ventricular global longitudinal strain (LV GLS)
Teo et al. [57]	2021	Meta-analysis	n = 9	337	Daytime bradycardia in OSA
Varounis et al. [58]	2014	Meta-analysis	n = 34	4852	24 h SBP
Vlachantoni et al. [59]	2013	Meta-analysis	n = 5	359	All arterial stiffness indices (pooled)
Xu et al. [60]	2015	Meta-analysis	n = 15	615	Flow-mediated dilation (FMD)
Yang et al. [61]	2023	Meta-analysis	n = 11	199	SBP change
Chen et al. [62]	2017	Systematic review	n = 29	1820	Change in carotid IMT
Aslan et al. [63]	2018	Meta-analysis	n = 14	5410	LVEF
Deng et al. [64]	2018	Systematic review	n = 9	3314	AF recurrence after catheter ablation
Benning et al. [65]	2025	Meta-analysis	n = 7	167	Office SBP
de Coelho Castro et al. [66]	2026	Meta-analysis	n = 9 (RCT = 5; observational = 4)	9610	LV GLS
Bratton et al. [67]	2015	Meta-analysis	n = 10 (RCT = 3; cohort = 7)	1217	SBP
Bratton et al. [68]	2014	Systematic review	n = NR	NR	SBP
da Silva Paulitsch et al. [69]	2019	Meta-analysis	n = 10	385	SBP
Fava et al. [70]	2014	Meta-analysis	n = 51	4888	SBP (Office/ABPM)
Lv et al. [71]	2024	Meta-analysis	n = 4	1219	SBP
Sun et al. [55]	2024	Meta-analysis	n = 52	10,104	24 h SBP
Sabry et al. [48]	2025	Systematic review	n = NR	NR	Blood pressure reduction (overall BP)
Shang et al. [52]	2022	Meta-analysis	n = NR	NR	24 h SBP
Sarvananda et al. [50]	2025	Systematic review	n = 8	731	Blood pressure reduction (overall BP)
Sun et al. [54]	2016	Meta-analysis	n = 12	1720	24 h SBP

Abbreviations: ABPM, ambulatory blood pressure monitoring; AF, atrial fibrillation; AHI, apnea–hypopnea index; BP, blood pressure; FMD, flow-mediated dilation; IMT, intima–media thickness; LVEF, left ventricular ejection fraction; LV GLS, left ventricular global longitudinal strain; mPAP, mean pulmonary arterial pressure; NR, not reported; OSA, obstructive sleep apnea; PAP, pulmonary arterial pressure; PASP, pulmonary artery systolic pressure; RCT, randomized controlled trial; SBP, systolic blood pressure.

**Table 2:** Methodological quality of included reviews (AMSTAR 2).

Study	Year	AMSTAR 2 Rating
Feng et al. [36]	2023	Low
Green et al. [37]	2021	Low
Guo et al. [27]	2016	Moderate
Guo et al. [38]	2018	Low
Han et al. [39]	2021	Critically low
Itaya et al. [40]	2025	Moderate
Khan et al. [41]	2017	Moderate
Kou et al. [42]	2022	Moderate
Labarca et al. [43]	2021	Critically low
Lei et al. [44]	2017	Moderate
Li et al. [45]	2023	Critically low
Montesi et al. [46]	2012	Low
Pengo et al. [47]	2020	Moderate
Sabry et al. [48]	2025	Critically low
Hu et al. [49]	2015	Moderate
Sarvananda et al. [50]	2025	Critically low
Schein et al. [51]	2014	Low
Shang et al. [52]	2022	Moderate
Sun et al. [72]	2013	Moderate
Sun et al. [53]	2014	Low
Sun et al. [54]	2016	Critically low
Sun et al. [55]	2024	Moderate
Tadic et al. [56]	2022	Moderate
Teo et al. [57]	2021	Moderate
Varounis et al. [58]	2014	Low
Vlachantoni et al. [59]	2013	Low
Xu et al. [60]	2015	Moderate
Yang et al. [61]	2023	Moderate
Chen et al. [62]	2017	Critically low
Aslan et al. [63]	2018	Low
Deng et al. [64]	2018	Critically low
Benning et al. [65]	2025	Moderate
de Coelho Castro et al. [66]	2026	Low
Bratton et al. [67]	2015	Moderate
Bratton et al. [68]	2014	Critically low
da Silva Paulitsch et al. [69]	2019	Moderate
Fava et al. [70]	2014	Moderate
Lv et al. [71]	2024	Moderate

**Table 3:** Summary of cardiovascular outcomes across included studies.

Outcome	Study	Effect Measure	Effect Size	95% CI	*p*-Value	Heterogeneity (I^2^)
Systolic blood pressure (RCT)	Green et al., 2021 [37]	MD	−4.8	−7.7 to −2.0	NR	84%
Diastolic blood pressure (RCT)	Green et al., 2021 [37]	MD	−3.0	−4.6 to −1.4	NR	62%
Systolic blood pressure (cohort)	Green et al., 2021 [37]	MD	−7.5	−11.7 to −3.3	NR	71%
Diastolic blood pressure (cohort)	Green et al., 2021 [37]	MD	−5.1	−8.0 to −2.3	NR	64%
24 h systolic blood pressure	Guo et al., 2016 [27]	MD	−2.03	−3.64 to −0.42	0.01	0%
24 h diastolic blood pressure	Guo et al., 2016 [27]	MD	−1.79	−2.89 to −0.68	0.001	0%
Nocturnal systolic blood pressure	Guo et al., 2016 [27]	MD	−4.39	−6.85 to −1.93	0.0005	34%
Nocturnal diastolic blood pressure	Guo et al., 2016 [27]	MD	−1.64	−2.88 to −0.40	0.009	0%
Daytime diastolic blood pressure	Guo et al., 2016 [27]	MD	−1.43	−2.67 to −0.19	0.02	0%
Left ventricular diastolic function (E/A ratio)	Feng et al., 2023 [36]	WMD	0.22	0.06 to 0.38	0.007	89.8%
Left ventricular ejection fraction (LVEF)	Han et al., 2021 [39]	MD	3.27	1.88 to 4.65	<0.00001	39%
Noradrenaline (RCT)	Green et al., 2021 [37]	SMD	−1.10	−1.53 to −0.56	NR	81%
Noradrenaline (cohort)	Green et al., 2021 [37]	SMD	−0.38	−0.53 to −0.24	NR	0%
Heart rate variability (LF)	Guo et al., 2018 [38]	SMD	−0.32	−0.62 to −0.01	0.043	49.6%
Heart rate variability (HF)	Guo et al., 2018 [38]	SMD	−0.08	−0.41 to 0.25	0.632	62.6%
LF/HF ratio	Guo et al., 2018 [38]	SMD	−0.30	−0.57 to −0.03	0.031	56.5%
BNP/NT-proBNP	Han et al., 2021 [39]	SMD	−0.60	−1.00 to −0.20	NR	NR
NYHA functional class	Han et al., 2021 [39]	MD	−0.71	−1.04 to −0.37	NR	NR
Body mass index (BMI)	Guo et al., 2016 [27]	MD	0.41	−0.11 to 0.93	0.12	0%

Abbreviations: BMI, body mass index; BNP, B-type natriuretic peptide; CI, confidence interval; HF, high frequency; LF, low frequency; LVEF, left ventricular ejection fraction; MD, mean difference; NR, not reported; NT-proBNP, N-terminal pro-B-type natriuretic peptide; NYHA, New York Heart Association; RCT, randomized controlled trial; SMD, standardized mean difference; WMD, weighted mean difference.

**Table 4:** Certainty of evidence for the main cardiovascular outcomes according to the GRADE framework.

Outcome	Evidence Source	Risk of Bias	Inconsistency	Indirectness	Imprecision	GRADE Certainty	Interpretation
Blood pressure (BP)	Meta-analyses of randomized and observational studies.	Not serious	Serious (I^2^ > 60%)	Not serious	Not serious	Moderate	Moderate-certainty evidence indicates that CPAP probably reduces systolic and diastolic blood pressure despite substantial statistical heterogeneity.
Heart rate variability (HRV)	Meta-analyses evaluating autonomic function.	Not serious	Serious	Not serious	Serious (small sample sizes)	Low	Low-certainty evidence suggests that CPAP may improve selected measures of heart rate variability; however, confidence in the estimate is limited because of heterogeneity and small sample sizes. Additional well-designed randomized trials are likely to influence the estimated effect.
Cardiovascular biomarkers	Meta-analyses reporting cardiovascular biomarkers.	Not serious	Serious	Not serious	Serious (limited reporting and small evidence base)	Low	Low-certainty evidence suggests that CPAP may improve selected cardiovascular biomarkers; however, confidence in the estimate is limited because of incomplete reporting and the small number of available studies.

Note. Certainty of evidence was assessed using the GRADE (Grading of Recommendations Assessment, Development and Evaluation) framework. I^2^ represents statistical heterogeneity across the included studies. Certainty ratings were downgraded when serious concerns regarding risk of bias, inconsistency, indirectness, imprecision, or publication bias were identified, in accordance with GRADE guidance.

## Data Availability

All data supporting the findings of this study are included within the article or are available from the corresponding author upon reasonable request.

## Supplementary Materials

The following supporting information can be downloaded at: https://www.mdpi.com/article/10.3390/medicina62081456/s1, Table S1: Complete Electronic Search Strategy.

## References

[1] Malhotra A., White D.P. (2002). Obstructive sleep apnoea. Lancet.

[2] Redline S., Yenokyan G., Gottlieb D.J., Shahar E., O’Connor G.T., Resnick H.E., Diener-West M., Sanders M.H., Wolf P.A., Geraghty E.M. (2010). Obstructive sleep apnea–hypopnea and incident stroke: The Sleep Heart Health Study. Am. J. Respir. Crit. Care Med..

[3] Platon A.L., Stelea C.G., Boișteanu O., Patrascanu E., Zetu I.N., Roșu S.N., Trifan V., Palade D.O. (2023). An update on obstructive sleep apnea syndrome—A literature review. Medicina.

[4] de Araujo Dantas A.B., Goncalves F.M., Martins A.A., Alves G.Â., Stechman-Neto J., Corrêa C.D.C., Santos R.S., Nascimento W.V., de Araujo C.M., Taveira K.V.M. (2023). Worldwide prevalence and associated risk factors of obstructive sleep apnea: A meta-analysis and meta-regression. Sleep Breath.

[5] Marshall N.S., Wong K.K., Liu P.Y., Cullen S.R., Knuiman M.W., Grunstein R.R. (2008). Sleep apnea as an independent risk factor for all-cause mortality: The Busselton Health Study. Sleep.

[6] Benjafield A.V., Ayas N.T., Eastwood P.R., Heinzer R., Ip M.S.M., Morrell M.J., Nunez C.M., Patel S.R., Penzel T., Pépin J.-L. (2019). Estimation of the global prevalence and burden of obstructive sleep apnoea: A literature-based analysis. Lancet Respir. Med..

[7] Senaratna C.V., Perret J.L., Lodge C.J., Lowe A.J., Campbell B.E., Matheson M.C., Hamilton G.S., Dharmage S.C. (2017). Prevalence of obstructive sleep apnea in the general population: A systematic review. Sleep Med. Rev..

[8] Somers V.K., White D.P., Amin R., Abraham W.T., Costa F., Culebras A., Daniels S., Floras J.S., Hunt C.E., Olson L.J. (2008). Sleep apnea and cardiovascular disease: An American Heart Association/American College of Cardiology Foundation Scientific Statement From the American Heart Association Council for High Blood Pressure Research Professional Education Committee, Council on Clinical Cardiology, Stroke Council, and Council on Cardiovascular Nursing In Collaboration With the National Heart, Lung, and Blood Institute National Center on Sleep Disorders Research (National Institutes of Health). Circulation.

[9] Mitra A.K., Bhuiyan A.R., Jones E.A. (2021). Association and risk factors for obstructive sleep apnea and cardiovascular diseases: A systematic review. Diseases.

[10] Ahmed A.M., Nur S.M., Xiaochen Y. (2023). Association between obstructive sleep apnea and resistant hypertension: Systematic review and meta-analysis. Front. Med..

[11] Hou H., Zhao Y., Yu W., Dong H., Xue X., Ding J., Xing W., Wang W. (2018). Association of obstructive sleep apnea with hypertension: A systematic review and meta-analysis. J. Glob. Health.

[12] Polecka A., Olszewska N., Danielski Ł., Olszewska E. (2023). Association between obstructive sleep apnea and heart failure in adults—A systematic review. J. Clin. Med..

[13] Prechaporn W., Hantrakul P., Ngamjarus C., Sukeepaisarnjaroen W., Sawanyawisuth K., Khamsai S. (2024). Pooled prevalences of obstructive sleep apnea and heart failure: A systematic review and meta-analysis. Heart Fail. Rev..

[14] Patel N., Donahue C., Shenoy A., Patel A., El-Sherif N. (2017). Obstructive sleep apnea and arrhythmia: A systemic review. Int. J. Cardiol..

[15] Mahajan S.K., Mahajan K., Sharma S. (2022). Obstructive sleep apnea and coronary artery disease: An unholy nexus or a holy alliance?. Lung India.

[16] Ooi E.L., Rajendran S. (2023). Obstructive sleep apnea in coronary artery disease. Curr. Probl. Cardiol..

[17] Almeneessier A.S., Alasousi N., Sharif M.M., Pandi-Perumal S.R., Hersi A.S., BaHammam A.S. (2017). Prevalence and predictors of arrhythmia in patients with obstructive sleep apnea. Sleep Sci..

[18] Hersi A.S. (2010). Obstructive sleep apnea and cardiac arrhythmias. Ann. Thorac. Med..

[19] May A.M., Van Wagoner D.R., Mehra R. (2017). OSA and cardiac arrhythmogenesis: Mechanistic insights. Chest.

[20] Zhang D., Ma Y., Xu J., Yi F. (2022). Association between obstructive sleep apnea (OSA) and atrial fibrillation (AF): A dose-response meta-analysis. Medicine.

[21] Polotsky V.Y., Patil S.P., Savransky V., Laffan A., Fonti S., Frame L.A., Steele K.E., Schweizter M.A., Clark J.M., Torbenson M.S. (2009). Obstructive sleep apnea, insulin resistance, and steatohepatitis in severe obesity. Am. J. Respir. Crit. Care Med..

[22] Fiedorczuk P., Stróżyński A., Olszewska E. (2020). Is the oxidative stress in obstructive sleep apnea associated with cardiovascular complications?—Systematic review. J. Clin. Med..

[23] Cammaroto G., Costa F., Ruiz M.V.G., Andò G., Vicini C., Montevecchi F., Galletti C., Galletti F., Valgimigli M. (2019). Obstructive sleep apnoea syndrome and endothelial function: Potential impact of different treatment strategies—Meta-analysis of prospective studies. Eur. Arch. Otorhinolaryngol..

[24] Schwab R.J., Badr S.M., Epstein L.J., Gay P.C., Gozal D., Kohler M., Lévy P., Malhotra A., Phillips B.A., Rosen I.M. (2013). An official American Thoracic Society statement: Continuous positive airway pressure adherence tracking systems. The optimal monitoring strategies and outcome measures in adults. Am. J. Respir. Crit. Care Med..

[25] Cheng Y., Ou Q., Chen B., Loffler K.A., McEvoy R.D., Xu Y., Wang Q., Lao M. (2023). The changes of AHI after long-term CPAP in patients with comorbid OSA and cardiovascular disease. Sleep Breath.

[26] Sánchez-de-la-Torre M., Gracia-Lavedan E., Benítez I.D., Sánchez-de-la-Torre A., Moncusí-Moix A., Torres G., Loffler K., Woodman R., Adams R., Labarca G. (2023). Adherence to CPAP treatment and the risk of recurrent cardiovascular events: A meta-analysis. JAMA.

[27] Guo J., Sun Y., Xue L.J., Huang Z.Y., Wang Y.S., Zhang L., Zhou G.-H., Yuan L.-X. (2016). Effect of CPAP therapy on cardiovascular events and mortality in patients with obstructive sleep apnea: A meta-analysis. Sleep Breath.

[28] Pengo M.F., Schwarz E.I., Barbé F., Cistulli P.A., Drager L.F., Fava C., Fuchs F.D., Ip M.S., Loffler K.A., Lui M.M. (2025). Effect of CPAP therapy on blood pressure in patients with obstructive sleep apnoea: A worldwide individual patient data meta-analysis. Eur. Respir. J..

[29] Javaid S.S., Obaid M.A., Noor T., Kaleem M., Shakil A., Haque S.U., Abbasi N.M., Saeed E.M., Manan S.M., Alam K.M. (2025). Impact of continuous positive airway pressure on cardiovascular health in patients with obstructive sleep apnea: A systematic review and meta-analysis. Cardiol. Rev..

[30] McEvoy R.D., Antic N.A., Heeley E., Luo Y., Ou Q., Zhang X., Mediano O., Chen R., Drager L.F., Liu Z. (2016). CPAP for prevention of cardiovascular events in obstructive sleep apnea. N. Engl. J. Med..

[31] Drager L.F., McEvoy R.D., Barbe F., Lorenzi-Filho G., Redline S. (2017). Sleep apnea and cardiovascular disease: Lessons from recent trials and need for team science. Circulation.

[32] Page M.J., McKenzie J.E., Bossuyt P.M., Boutron I., Hoffmann T.C., Mulrow C.D., Shamseer L., Tetzlaff J.M., Akl E.A., Brennan S.E. (2021). The PRISMA 2020 statement: An updated guideline for reporting systematic reviews. BMJ.

[33] Aromataris E., Fernandez R., Godfrey C.M., Holly C., Khalil H., Tungpunkom P. (2015). Summarizing systematic reviews: Methodological development, conduct and reporting of an umbrella review approach. Int. J. Evid. Based Healthc..

[34] Shea B.J., Reeves B.C., Wells G., Thuku M., Hamel C., Moran J., Moher D., Tugwell P., Welch V., Kristjansson E. (2017). AMSTAR 2: A critical appraisal tool for systematic reviews that include randomised or non-randomised studies of healthcare interventions, or both. BMJ.

[35] Guyatt G.H., Oxman A.D., Vist G.E., Kunz R., Falck-Ytter Y., Alonso-Coello P., Schünemann H.J. (2008). GRADE: An emerging consensus on rating quality of evidence and strength of recommendations. BMJ.

[36] Feng J., Li K., Luo W., Xie F., Li M., Wu Y. (2023). Effect of continuous positive pressure ventilation on left ventricular diastolic function E/A ratio in patients with obstructive sleep apnea: A meta-analysis. Sleep Breath.

[37] Green M., Ken-Dror G., Fluck D., Sada C., Sharma P., Fry C.H., Han T.S. (2021). Meta-analysis of changes in the levels of catecholamines and blood pressure with continuous positive airway pressure therapy in obstructive sleep apnea. J. Clin. Hypertens..

[38] Guo W., Lv T., She F., Miao G., Liu Y., He R., Xue Y., Nu N.K., Yang J., Li K. (2018). The impact of continuous positive airway pressure on heart rate variability in obstructive sleep apnea patients during sleep: A meta-analysis. Heart Lung.

[39] Han Y.-C., Shen Z.-J., Zhang S.-Y., Gao P., Xiang R.-L., Qian H., Xie H.-Z. (2021). Efficacy of adaptive servo-ventilation and continuous positive airway pressure treatment in chronic heart failure with sleep-disordered breathing: A systematic review and meta-analysis. Heart Fail. Rev..

[40] Itaya E.D., Ternes C.M.P., Maher T., Fernandes A.A.D., Rocha A.V., Wippel C., Rivera A., Locke A.H., d’Avila A. (2025). Efficacy of continuous positive airway pressure on atrial fibrillation recurrence after catheter ablation in patients with obstructive sleep apnea: A systematic review and meta-analysis. J. Interv. Card. Electrophysiol..

[41] Khan S.U., Duran C.A., Rahman H., Lekkala M., Saleem M.A., Kaluski E. (2018). A meta-analysis of continuous positive airway pressure therapy in prevention of cardiovascular events in patients with obstructive sleep apnoea. Eur. Heart J..

[42] Kou C., Zhao X., Lin X., Fan X., Wang Q., Yu J. (2022). Effect of different treatments for obstructive sleep apnoea on blood pressure. J. Hypertens..

[43] Labarca G., Schmidt A., Dreyse J., Jorquera J., Enos D., Torres G., Barbé F. (2021). Efficacy of continuous positive airway pressure (CPAP) in patients with obstructive sleep apnea (OSA) and resistant hypertension (RH): Systematic review and meta-analysis. Sleep Med. Rev..

[44] Lei Q., Lv Y., Li K., Ma L., Du G., Xiang Y., Li X. (2017). Effects of continuous positive airway pressure on blood pressure in patients with resistant hypertension and obstructive sleep apnea: A systematic review and meta-analysis of six randomized controlled trials. J. Bras. Pneumol..

[45] Li F., He C.-J., Ding C.-H., Wang R.-X., Li H. (2023). Continuous positive airway pressure therapy might be an effective strategy on reduction of atrial fibrillation recurrence after ablation in patients with obstructive sleep apnea: Insights from the pooled studies. Front. Neurol..

[46] Montesi S.B., Edwards B.A., Malhotra A., Bakker J.P. (2012). The effect of continuous positive airway pressure treatment on blood pressure: A systematic review and meta-analysis of randomized controlled trials. J. Clin. Sleep Med..

[47] Pengo M.F., Soranna D., Giontella A., Perger E., Mattaliano P., Schwarz E.I., Lombardi C., Bilo G., Zambon A., Steier J. (2020). Obstructive sleep apnoea treatment and blood pressure: Which phenotypes predict a response? A systematic review and meta-analysis. Eur. Respir. J..

[48] Sabry M., Gundala C., Hamad M., Tummala N., Hamid A.A., Mohamed A.E., Zia U., Hassan A.M., Essani B., Abid M. (2025). Impact of sleep apnea treatment with continuous positive airway pressure (CPAP) on blood pressure control in resistant hypertension: A systematic review and meta-analysis. Cureus.

[49] Hu X., Fan J., Chen S., Yin Y., Zrenner B. (2015). The role of continuous positive airway pressure in blood pressure control for patients with obstructive sleep apnea and hypertension: A meta-analysis of randomized controlled trials. J. Clin. Hypertens..

[50] Sarvananda S., Earnshaw S., Hughes I., Sivakumaran P., Sriram K.B. (2025). Effect of positive airway pressure treatment on pulmonary artery pressure in obstructive sleep apnoea and/or obesity hypoventilation syndrome with pulmonary hypertension: A systematic review and meta-analysis. Intern. Med. J..

[51] Schein A.S., Kerkhoff A.C., Coronel C.C., Plentz R.D.M., Sbruzzi G. (2014). Continuous positive airway pressure reduces blood pressure in patients with obstructive sleep apnea; A systematic review and meta-analysis with 1000 patients. J. Hypertens..

[52] Shang W., Zhang Y., Liu L., Chen F., Wang G., Han D. (2022). Benefits of continuous positive airway pressure on blood pressure in patients with hypertension and obstructive sleep apnea: A meta-analysis. Hypertens. Res..

[53] Sun X., Luo J., Xiao Y. (2014). Continuous positive airway pressure is associated with a decrease in pulmonary artery pressure in patients with obstructive sleep apnoea: A meta-analysis. Respirology.

[54] Sun Y., Huang Z.Y., Sun Q.R., Qiu L.P., Zhou T.T., Zhou G.H. (2016). CPAP therapy reduces blood pressure for patients with obstructive sleep apnoea: An update meta-analysis of randomized clinical trials. Acta Cardiol..

[55] Sun L., Chang Y.F., Wang Y.F., Xie Q.X., Ran X.Z., Hu C.Y., Luo B., Ning B. (2024). Effect of Continuous Positive Airway Pressure on Blood Pressure in Patients with Resistant Hypertension and Obstructive Sleep Apnea: An Updated Meta-analysis. Curr. Hypertens. Rep..

[56] Tadic M., Gherbesi E., Faggiano A., Sala C., Carugo S., Cuspidi C. (2022). The impact of continuous positive airway pressure on cardiac mechanics: Findings from a meta-analysis of echocardiographic studies. J. Clin. Hypertens..

[57] Teo Y.H., Han R., Leong S., Teo Y.N., Syn N.L., Wee C.F., Tan B.K.J., Wong R.C.C., Chai P., Kojodjojo P. (2022). Prevalence, types and treatment of bradycardia in obstructive sleep apnea—A systematic review and meta-analysis. Sleep Med..

[58] Varounis C., Katsi V., Kallikazaros I.E., Tousoulis D., Stefanadis C., Parissis J., Lekakis J., Siristatidis C., Manolis A.J., Makris T. (2014). Effect of CPAP on blood pressure in patients with obstructive sleep apnea and resistant hypertension: A systematic review and meta-analysis. Int. J. Cardiol..

[59] Vlachantoni I.-T., Dikaiakou E., Antonopoulos C.N., Stefanadis C., Daskalopoulou S.S., Petridou E.T. (2013). Effects of continuous positive airway pressure (CPAP) treatment for obstructive sleep apnea in arterial stiffness: A meta-analysis. Sleep Med. Rev..

[60] Xu H., Wang Y., Guan J., Yi H., Yin S. (2015). Effect of CPAP on endothelial function in subjects with obstructive sleep apnea: A meta-analysis. Respir. Care.

[61] Yang D., Li L., Dong J., Yang W., Liu Z. (2023). Effects of continuous positive airway pressure on cardiac events and metabolic components in patients with moderate to severe obstructive sleep apnea and coronary artery disease: A meta-analysis. J. Clin. Sleep Med..

[62] Chen L.D., Lin L., Lin X.J., Ou Y.W., Wu Z., Ye Y.M., Xu Q.-Z., Huang Y.-P., Cai Z.-M. (2017). Effect of continuous positive airway pressure on carotid intima-media thickness in patients with obstructive sleep apnea: A meta-analysis. PLoS ONE.

[63] Aslan G., Afsar B., Siriopol D., Kanbay A., Sal O., Benli C., Okcuoglu J., Covic A., Kanbay M. (2018). Cardiovascular effects of continuous positive airway pressure treatment in patients with obstructive sleep apnea: A meta-analysis. Angiology.

[64] Deng F., Raza A., Guo J. (2018). Treating obstructive sleep apnea with continuous positive airway pressure reduces risk of recurrent atrial fibrillation after catheter ablation: A meta-analysis. Sleep Med..

[65] Benning L., Herzig J.J., Mollet M.S., Bradicich M., Pengo M.F., Ulrich S., Schwarz E.I. (2025). Effects of CPAP on blood pressure parameter across different severities of obstructive sleep apnoea: A meta-analysis. J. Sleep Res..

[66] de Coelho Castro P., Prizão V.M., Erzinger G., Leite F.V.L., Barbosa L.M., de Lucena L.A., Castro R.S.A., Girardi A.C.C., Reichert C.O., Drager L.F. (2026). Efficacy of continuous positive airway pressure on cardiac remodeling and ventricular function in obstructive sleep apnea: A systematic review and updated meta-analysis of speckle-tracking echocardiography. J. Hypertens..

[67] Bratton D.J., Gaisl T., Wons A.M., Kohler M. (2015). CPAP vs mandibular advancement devices and blood pressure in patients with obstructive sleep apnea: A systematic review and meta-analysis. JAMA.

[68] Bratton D.J., Stradling J.R., Barbé F., Kohler M. (2014). Effect of CPAP on blood pressure in patients with minimally symptomatic obstructive sleep apnoea: A meta-analysis using individual patient data from four randomised controlled trials. Thorax.

[69] da Silva Paulitsch F., Zhang L. (2019). Continuous positive airway pressure for adults with obstructive sleep apnea and cardiovascular disease: A meta-analysis of randomized trials. Sleep Med..

[70] Fava C., Dorigoni S., Dalle Vedove F., Danese E., Montagnana M., Guidi G.C., Narkiewicz K., Minuz P. (2014). Effect of CPAP on blood pressure in patients with OSA/hypopnea: A systematic review and meta-analysis. Chest.

[71] Lv M., Mao J., Wang S., Zhang C., Qian C., Zhu R., Xiong S., Zhang Y., Guo L. (2024). Effect of continuous positive airway pressure on cardiometabolic risk factors in patients with obstructive sleep apnea: A systematic review and meta-analysis. Respir. Med..

[72] Sun H., Shi J., Li M., Chen X. (2013). Impact of continuous positive airway pressure treatment on left ventricular ejection fraction in patients with obstructive sleep apnea: A meta-analysis of randomized controlled trials. PLoS ONE.

[73] Peker Y., Glantz H., Eulenburg C., Wegscheider K., Herlitz J., Thunström E. (2016). Effect of positive airway pressure on cardiovascular outcomes in coronary artery disease patients with nonsleepy obstructive sleep apnea: The RICCADSA randomized controlled trial. Am. J. Respir. Crit. Care Med..

