# Supplementary material for: Effects of Continuous Positive Airway Pressure on Surrogate, Intermediate, and Functional Cardiovascular Outcomes in Obstructive Sleep Apnea: An Umbrella Review of Systematic Reviews and Meta-Analyses
Source: Medicina (Kaunas). 2026 Jul 27;62(8):1456. doi: 10.3390/medicina62081456 (PMC13514981; doi:10.3390/medicina62081456)
Supplement: Supplementary file 1 [file medicina-62-01456-s001.zip › medicina-4390814-supplementary.pdf]

Supplementary Table S1. Complete Electronic Search Strategy

Search period: Database inception to 28 February 2026

Limits: Humans; English language; Publication years: 2010–2026

PubMed (MEDLINE)

```
Database: PubMed (MEDLINE)

Coverage: Database inception to 28 February 2026

Search concept 1 – Obstructive Sleep Apnea
("Sleep Apnea, Obstructive"[MeSH Terms] OR "sleep apnea
obstructive"[Title/Abstract] OR "obstructive sleep apnea"[Title/Abstract] OR
"obstructive sleep apnoea"[Title/Abstract] OR OSA[Title/Abstract] OR
OSAHS[Title/Abstract] OR OSAS[Title/Abstract] OR OSAH[Title/Abstract] OR
SAS[Title/Abstract] OR SAHS[Title/Abstract] OR SHS[Title/Abstract] OR
SDB[Title/Abstract] OR "sleep apnea hypopnea syndrome"[Title/Abstract] OR
"sleep apnoea hypopnoea syndrome"[Title/Abstract] OR "obstructive sleep apnea
hypopnea syndrome"[Title/Abstract] OR "obstructive sleep apnea
syndrome"[Title/Abstract] OR "obstructive sleep apnoea
syndrome"[Title/Abstract] OR "sleep disordered breathing"[Title/Abstract])

Search concept 2 – CPAP
("Continuous Positive Airway Pressure"[MeSH Terms] OR "continuous positive
airway pressure"[Title/Abstract] OR CPAP[Title/Abstract] OR
nCPAP[Title/Abstract] OR "nasal CPAP"[Title/Abstract] OR "nasal continuous
positive airway pressure"[Title/Abstract] OR "Airway Pressure Release
Ventilation"[Title/Abstract] OR APRV[Title/Abstract] OR "Biphasic Positive
Airway Pressure"[Title/Abstract] OR "Bilevel Positive Airway
Pressure"[Title/Abstract] OR BiPAP[Title/Abstract] OR "Non-Invasive Positive
Pressure Ventilation"[Title/Abstract] OR NIPPV[Title/Abstract] OR
NPPV[Title/Abstract] OR "Positive Pressure Ventilation"[Title/Abstract] OR
"Non-Invasive Assisted Ventilation"[Title/Abstract] OR NIAV[Title/Abstract] OR
"Positive Airway Pressure"[Title/Abstract])

Search concept 3 – Cardiovascular Outcomes
("Cardiovascular Diseases"[MeSH] OR "Heart Diseases"[MeSH] OR "Myocardial
Infarction"[MeSH] OR "Hypertension"[MeSH] OR "Coronary Disease"[MeSH] OR
"Coronary Artery Disease"[MeSH] OR "Myocardial Ischemia"[MeSH] OR
"Microvascular Angina"[MeSH] OR "Heart Failure"[MeSH] OR "Cerebrovascular
Disorders"[MeSH] OR "Stroke"[MeSH] OR "Ischemic Stroke"[MeSH] OR
"cardiovascular risk"[Title/Abstract] OR "cardiovascular
outcome"[Title/Abstract] OR hypertension[Title/Abstract] OR "coronary artery
disease"[Title/Abstract] OR "congestive heart failure"[Title/Abstract] OR
"cardiac arrhythmias"[Title/Abstract] OR stroke[Title/Abstract] OR
"cardiovascular events"[Title/Abstract] OR "cardiovascular
death"[Title/Abstract] OR "cardiovascular mortality"[Title/Abstract] OR
cardiovascular[Title/Abstract] OR "coronary disease"[Title/Abstract] OR
"coronary heart disease"[Title/Abstract] OR myocardi*[Title/Abstract] OR
angina*[Title/Abstract] OR "ischemic cardiomyopathy"[Title/Abstract] OR
"ischemic heart disease"[Title/Abstract] OR "ischemic
cardiopathy"[Title/Abstract] OR "percutaneous coronary
```

intervention"[Title/Abstract] OR "coronary artery bypass grafting"[Title/Abstract] OR PCI[Title/Abstract] OR "heart attack"[Title/Abstract] OR CHD[Title/Abstract] OR CAD[Title/Abstract] OR "heart failure"[Title/Abstract] OR "atrial function"[Title/Abstract] OR "ventricular function"[Title/Abstract] OR "cardiac function"[Title/Abstract] OR "cardiac structure"[Title/Abstract] OR cerebrovascular[Title/Abstract] OR "cerebral vascular"[Title/Abstract] OR "white matter hyperintense lesions"[Title/Abstract] OR WMHI[Title/Abstract] OR CVA[Title/Abstract] OR "ischemic encephal*"[Title/Abstract] OR hemorrhag*[Title/Abstract] OR "transient ischemic attack"[Title/Abstract] OR TIA[Title/Abstract] OR "cerebral ischemia"[Title/Abstract] OR "cerebral small vessel disease"[Title/Abstract] OR hypertens*[Title/Abstract] OR "blood pressure"[Title/Abstract] OR "peripheral arterial"[Title/Abstract] OR mortality[Title/Abstract] OR death[Title/Abstract] OR infarction*[Title/Abstract] OR atherosclerosis[Title/Abstract])

Final search:
(Search concept 1) AND (Search concept 2) AND (Search concept 3)

Embase

Database: Embase

Coverage: Database inception to 28 February 2026

Search concept 1
('obstructive sleep apnea'/exp OR 'obstructive sleep apnea' OR 'obstructive sleep apnoea' OR osa OR osas OR osahs OR 'sleep disordered breathing' OR sdb OR 'upper airway resistance syndrome')

Search concept 2
('continuous positive airway pressure'/exp OR 'continuous positive airway pressure' OR 'cpap'/exp OR cpap OR 'nasal continuous positive airway pressure' OR ncpap OR 'airway pressure release ventilation' OR aprv OR 'biphasic positive airway pressure' OR 'bilevel positive airway pressure' OR bipap OR 'non-invasive positive pressure ventilation' OR nippv OR nppv OR 'positive pressure ventilation'/exp OR 'positive pressure ventilation' OR 'non-invasive assisted ventilation' OR niav OR niv OR 'positive airway pressure'/exp OR 'positive airway pressure')

Search concept 3
('cardiovascular death':ab,ti OR 'cardiovascular mortality':ab,ti OR cardiovascular disease/ OR 'hypotension'/exp OR 'coronary artery disease'/exp OR 'heart arrhythmia'/exp OR 'heart atrium function'/exp OR 'heart failure'/exp OR 'pulmonary hypertension'/exp OR 'heart ventricle function'/exp OR 'heart left ventricle function'/exp OR 'hypertension'/exp OR 'atherosclerosis'/exp OR 'cardiovascular system'/exp)

Final search:
(Search concept 1) AND (Search concept 2) AND (Search concept 3)

Cochrane Library

Database: Cochrane Library

Coverage: Database inception to 28 February 2026

Search concept 1
MeSH descriptor: [Sleep Apnea, Obstructive] explode all trees OR MeSH descriptor: [Sleep Apnea Syndromes] explode all trees OR (obstruct* or sleep) and (apnea* or apnoea*) OR obstruct* and (hypopnea* or hypopnoea* or hypoapnea* or hypoapnoea*) OR obstructive sleep apnea OR obstructive sleep apnoea OR OSA OR OSAS OR OSAHS OR upper airway resistance syndrome OR sleep-disordered breathing OR SDB

Search concept 2
("Continuous Positive Airway Pressure" OR CPAP OR "Nasal Continuous Positive Airway Pressure" OR nCPAP OR "Airway Pressure Release Ventilation" OR APRV OR "Biphasic Positive Airway Pressure" OR "Bilevel Positive Airway Pressure" OR BiPAP OR "Non-Invasive Positive Pressure Ventilation" OR NIPPV OR NPPV OR "Positive Pressure Ventilation" OR "Non-Invasive Assisted Ventilation" OR NIAV OR NIV OR "Positive Airway Pressure")

Search concept 3
MeSH descriptor: [Cardiovascular Diseases] explode all trees OR cardiovascular diseases OR coronary disease OR coronary heart disease OR coronary artery disease OR myocardi* OR angina* OR ischemic cardiomyopathy OR ischemic heart disease OR ischemic cardiopathy OR percutaneous coronary intervention OR coronary artery bypass grafting OR PCI OR heart attack OR CHD OR CAD OR heart failure OR atrial function OR ventricular function OR cardiac function OR cardiac structure OR hypertens* OR blood pressure OR peripheral arterial OR mortality OR death OR infarction* OR atherosclerosis

Final search:
(Search concept 1) AND (Search concept 2) AND (Search concept 3)
